# A Comprehensive Phylogenetic Analysis of SARS-CoV-2: Utilizing a Novel and Convenient In-House RT-PCR Method for Characterization without Virus Culture and BSL-3 Facilities

**DOI:** 10.3390/v15071562

**Published:** 2023-07-16

**Authors:** Yen-Ju Chen, Jason C. Huang, Ching-Ping Yang, Kuo-Feng Hsu, Hsin-Fu Liu

**Affiliations:** 1 Research Assistant Center, Tainan Municipal Hospital (Managed by Show Chwan Medical Care Corporation), Tainan 701033, Taiwan; 2a7101@tmh.org.tw; 2 Department of Biotechnology and Laboratory Science in Medicine, National Yang Ming Chiao Tung University, Taipei 112304, Taiwan; jasonhuang@nycu.edu.tw; 3 Department of Medical Technology, Jenteh Junior College of Medicine, Nursing and Management, Miaoli 356006, Taiwan; d49407012@ym.edu.tw; 4 Department of Surgery, Tri-Service General Hospital, National Defense Medical Center, Taipei 114202, Taiwan; 5 Department of Medical Research, MacKay Memorial Hospital, Taipei 25169, Taiwan; 6 Institute of Biomedical Sciences, MacKay Medical College, New Taipei City 252005, Taiwan

**Keywords:** COVID-19, SARS-CoV-2, clinical specimens, in-house reverse transcription PCR, amino acid usage patterns

## Abstract

We developed a convenient method for amplifying the complete SARS-CoV-2 sequence using in-house RT-PCR without virus culture. Forty-one stored throat swabs and blood specimens were collected from eight SARS-CoV-2 infections at multiple time points. Total RNA was extracted using the QIAamp viral RNA mini kit and pooled for higher RNA levels. Only those positive specimens by commercial real-time RT-PCR (RT-qPCR) were selected and amplified by in-house RT-PCR for complete sequences, followed by sequencing. Phylogenetic trees and exploratory analyses were performed using MEGA 11 and Simplot 3.5.1 software. Swab samples had significantly higher total RNA concentrations than plasma (*p* < 0.01). Positive results were found mainly in swabs, but one was found in plasma. Successful gene amplification depended on Ct values (Ct < 38). A non-synonymous substitution was found in ORF1ab/Nsp3 (at NC045512.2 position 6312, C to A) and most spike protein mutations occurred in the S1 subunit (residues 14–685). The proposed method is time-saving and reliable for rapid genomic analysis. Increasing sample volume and pooling them for RNA extraction increases RNA concentration without culture. Combining nucleotide sequences from specific variable regions of the genome is more efficient than conventional methods.

## 1. Introduction

Among the various coronaviruses (CoVs) discovered so far, three of them—namely, SARS-CoV, MERS-CoV, and SARS-CoV-2—are highly pathogenic and cause endemic CoV diseases [1,2]. Human-infecting coronaviruses, like SARS-CoV and SARS-CoV-2, primarily spread through respiratory droplets, airborne routes, or direct contact [3]. The COVID-19 pandemic, an infectious disease caused by SARS-CoV-2, has resulted in catastrophic mortality and morbidity worldwide. As of 16 June 2023, more than 767 million people had been confirmed to have had SARS-CoV-2 infection, with 6,943,390 deaths attributed to COVID-19 [4]. Since the initial COVID-19 outbreak in China [5,6], Taiwan has demonstrated effective measures in preventing the spread of COVID-19 [7,8], particularly before March 2022. Currently, the Taiwan Central Epidemic Command Center (Taiwan CECC) has officially implemented a relaxation of its epidemic prevention measures on 1 May 2023, in response to a slight improvement in the epidemic situation.

The emergence of COVID-19 has led to global demand for precise diagnostic assays. Utilizing viral culture for immediate diagnosis is impractical as it typically takes at least three days for SARS-CoV-2 to cause observable cytopathic effects (CPE) in specific cell lines [9]. Moreover, culture isolates require biosafety level 3 (BSL-3) facilities that are not commonly available in healthcare institutions [10,11]. Additionally, the production of antibodies takes at least a few weeks in the case of IgM and even more for IgG. This makes the search for antibodies not very useful for early diagnosis of infection with viruses such as SARS-CoV. Serum antibody and antigen detection tests have not yet undergone thorough validation, and there may be instances of cross-reactivity with SARS-CoV [12]. Given these limitations, the most widely employed in vitro validation method with clinical specimens is real-time reverse transcription PCR (RT-qPCR) [13,14,15], which is considered the gold standard for confirming infections. The ORF1ab, S, E, and N genes have been commonly selected and designed as amplification targets for nucleic acid tests [13,16]. Initially, the Centers for Disease Control and Prevention (CDC) assay focused on three regions of the viral gene (E, RNA-dependent RNA polymerase (RdRp), and N) for detection and utilized the human RNase P gene as an internal control [16]. Moreover, a cycle threshold (Ct) value of less than 40 has been clinically reported as indicative of a positive result for RT-qPCR [14,15].

The number of SARS-CoV-2 whole genome sequences obtained through next-generation sequencing (NGS) is increasing rapidly daily. Currently, there are 1,956,955 complete SARS-CoV-2 sequences available on the NCBI website (https://www.ncbi.nlm.nih.gov/sars-cov-2/, accessed on 15 June 2023). It is crucial to perform genetic analysis using complete sequences due to concerns about molecular adaptation and recombination events. However, there is potential for simplification. In this study, we have developed a novel and convenient in-house reverse transcription PCR (RT-PCR) method to amplify the entire SARS-CoV-2 genome sequence. The significant advantage of this approach is that it does not require virus culture or BSL-3 facilities. Certain regions of the SARS-CoV-2 genome are more conserved than others, leading researchers to primarily focus on the S and N proteins, which have a higher tendency to evolve. To reduce the costs associated with variant detection methods, we have introduced several variable regions (v1–v5) as substitutes for whole genome amplification. The majority of substitutions occur within the ORF1ab gene, specifically in the segment responsible for encoding Nsp3. Moreover, combining nucleotide sequences from specific variable regions of the entire genome has proven to be more effective than conventional methods.

## 2. Materials and Methods

### 2.1. Subjects Acquisition and Processing

Stored throat swabs (n = 7) and blood samples (n = 34) were taken from 8 confirmed patients with COVID-19 (#P1, #P2, and #P7–#P12) at the National Yang Ming Chiao Tung University (NYCU) Hospital. This study received approval from both the Institutional Review Board of NYCU and NYCU Hospital, with IRB numbers YM110042E and 2021D002.

### 2.2. RNA Extraction, PCR Conditions, and Sequencing

The CDC and the World Health Organization (WHO) recommend that clinical samples, including both confirmed and suspected COVID-19 specimens, can be safely handled in BSL-2 facilities for regular diagnostic purposes [17,18]. To ensure the well-being of the personnel responsible for handling these samples, we performed RNA extraction from throat swabs at the Department of Laboratory Medicine in NYCU Hospital. This facility is equipped with a BSL-2 plus laboratory that incorporates negative pressure. Similarly, the RNA extraction from blood specimens was conducted at NYCU using their BSL-2 laboratory.

RNA was extracted from throat swabs and plasma samples using the QIAamp Viral RNA Mini Kit (Qiagen, Hilden, Germany). To enhance the yield of viral RNA in clinical samples, we pooled double the volume into a single extraction column, followed by a series of wash procedures. The eluted RNA was then treated with 60 μL of buffer AVE (refer to Supplementary Materials). For assessing the presence of SARS-CoV-2 in nasopharyngeal swabs, a commercial RT-qPCR assay (Lightcycler^®^ multiplex RNA virus master, Roche, Basel, Switzerland) was performed following the manufacturer’s instructions, and only specimens with positive results were selected for in-house RT-PCR amplification targeting nearly full-length sequences.

For detailed information on the in-house RT-PCR primers and PCR conditions, please refer to the Supplement (Table S1). The PCR products were purified by gel electrophoresis and subjected to DNA sequencing using an ABI PRISM 3700 DNA analyzer (Applied Biosystems, Woburn, MA, USA) at the Genome Research Center of NYCU. The partial sequences (OM250117-OM250118) obtained from this study have been deposited in GenBank.

### 2.3. Phylogenetic Analysis

Both GISAID (https://gisaid.org/, accessed on 12 August 2021) and the NCBI website have compiled a substantial number of complete sequences of SARS-CoV-2. In this study, we obtained the SARS-CoV-2 nucleotide sequences as reference sequences from the NCBI SARS-CoV-2 Resources (https://www.ncbi.nlm.nih.gov/sars-cov-2/, accessed on 12 August 2021) to facilitate resource accessibility. For the analysis of nucleotide signatures and amino acid usage patterns, we selected representative strains of COVID-19/SARS-CoV-2 with complete sequences from neighboring countries near Taiwan or countries frequently visited by Taiwanese individuals. As the cases we collected belonged to the initial stages of SARS-CoV-2 infections in Taiwan, we conducted comparisons with viral strains responsible for multiple waves of outbreaks in Taiwan (n = 18) and neighboring countries (n = 33). SARS-CoV-2 isolates from related countries, including AUT (n = 3), BRA (n = 4), CHN (n = 5), ESP (n = 12), FIN (n = 3), HKG (n = 13), IND (n = 10), ITA (n = 7), JPN (n = 8), KOR (n = 7), SWE (n = 3), USA (n = 7), and ZAF (n = 2). In total, we analyzed 102 complete SARS-CoV-2 sequences in the final dataset. The nucleotide sequence of the Wuhan-Hu-1 reference strain (accession number: NC045512; version: NC045512.2) was used as a prototype in this study.

Multiple sequence alignment was performed using ClustalW [19]. SimPlot 3.5.1 [20] was employed for bootscanning. The MEGA 11 software [21] and Phylip 3.6 [22] were utilized to construct phylogenetic trees. The best-fit nucleotide substitution model was determined using model selection in MEGA 11. The model with the lowest Bayesian information criterion (BIC) score was considered the most suitable. The presence of variations in evolutionary rates among sites can be accounted for by incorporating a discrete Gamma distribution (+G) with 5 rate categories and by considering a certain proportion of sites as evolutionarily invariable (+I). Positions that contained gaps and missing data were excluded from the analysis (using the complete deletion option). This study employed three datasets with distinct lengths for evolutionary analysis: the full length (~29.8 kb), v1–v5 (~6.5 kb), and v3 only (~1.4 kb).

In the case of full-length sequences, the maximum likelihood (ML) method employed the GTR + G + I substitution model, while TN93 + G was utilized for calculating the evolutionary distance in the neighbor-joining tree (NJ). However, when assembling the 5 variant regions (v1–v5) and using only v3 for the analysis, both ML and NJ trees were constructed by using the T92 + G and T92, respectively. Bootstrap analysis with 1000 replicates was conducted to assess the robustness of both NJ and ML trees generated through the conventional strategy. Bootstrap values (≥70%) were considered significant indicators of cluster significance.

### 2.4. Statistical Analysis

All analyses were conducted using SAS 9.4 software (SAS Institute, Cary, NC, USA). The baseline characteristics of all patients at admission were assessed using *t*-tests for continuous variables and chi-square tests for categorical data. A two-sided *p*-value of less than 0.05 was considered statistically significant. When conducting genome-wide studies, a large number of hypothesis tests are often performed simultaneously. We utilized the false discovery rate (FDR) and its analog, the q-value, to maximize the identification of significant comparisons while controlling the false positive rate. We calculated a *p*-value and setting our alpha level to 0.05 to ensure that the probability of a type I error, or a false positive, is below 5%.

## 3. Results

### 3.1. Epidemiological Information

**Figure 1:**
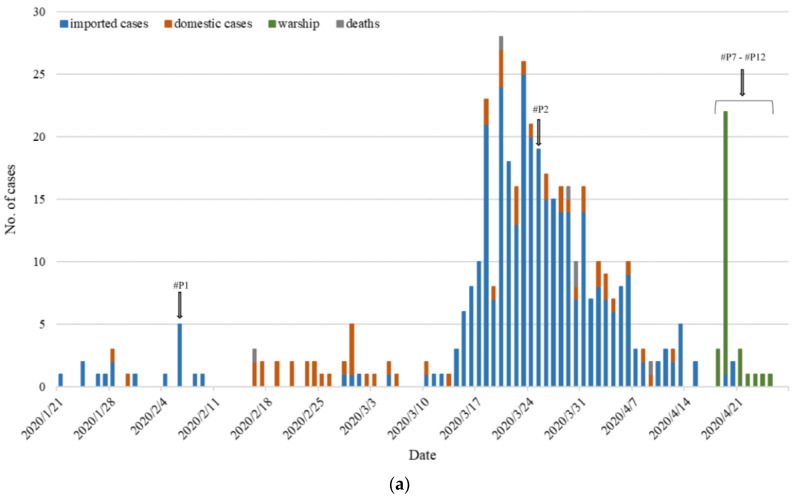

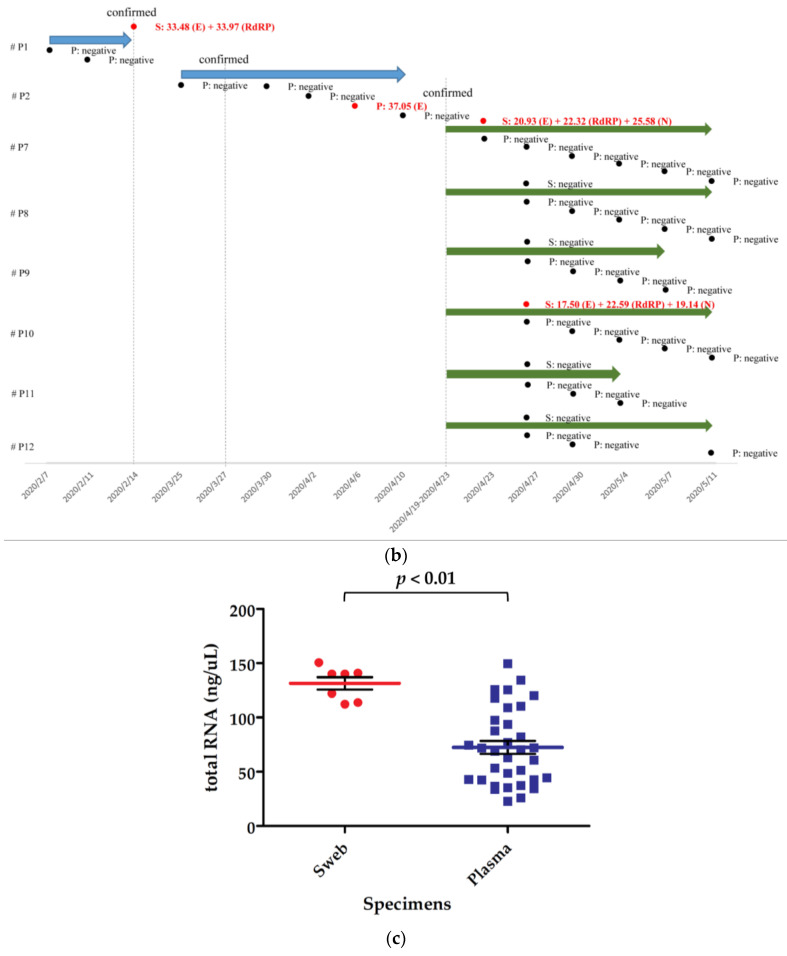
COVID-19 epidemic in Taiwan. (**a**) At the end of April 2020, 429 Taiwanese people were confirmed to have SARS-CoV-2 infection, and 6 deaths were due to COVID-19. Data sources are available from the Taiwan CDC. (**b**) Forty-one stored samples were collected from 8 confirmed patients with COVID-19, 9.8% (4/41) of them with positive reactions to the detection of RT-qPCR. Clinical specimen S means throat swab and P means plasma. (**c**) In general, concentrated extraction of total RNA in swabs and plasma. The straight line indicates the mean.

Figure 1a illustrates that by the end of April 2020, there had been 429 reported cases of SARS-CoV-2 infection in Taiwan, resulting in six deaths from COVID-19. Among these cases, eight early Taiwanese infections (#P1, #P2, and #P7–#P12) were identified and recruited from NYCU Hospital. The epidemiological information for these patients is as follows: Patient 1 (#P1) was a Taiwanese businessman residing in Wuhan, who returned to Taiwan for the Lunar New Year holidays and was confirmed on February 6. Patient 2 (#P2) was a student studying in the United States and was diagnosed with COVID-19 on March 27 upon her return to Taiwan. Patients 7–12 (#P7–#P12) were Navy crew members aboard the Panshi Fast Combat Support Ship and tested positive for the virus. Since their infections occurred while on the vessels and subsequently tested positive during the isolation period, their confirmed dates were approximately set between 19 April and 23 April (refer to Figure 1a). Although clinical samples were not collected from some patients at the onset of symptoms, nucleic acid detection was still performed using the concentrated extraction method of total RNA (Figure 1b and Table 1).

### 3.2. Both Swab and Plasma Specimens Can Be Performed and Detected by Commercial RT-qPCR

The mean total RNA concentrations in throat swabs and plasma samples were 131.4 ng/μL and 72.4 ng/μL, respectively. The total RNA concentration in swab samples was significantly higher than that in plasma (*p* < 0.01, Figure 1c). The RT-qPCR results are presented in Table 1. The RNase P gene, a normal human gene used as a housekeeping gene, was detected in almost all of the samples (39/41, 95.1%), ensuring the quality of sample collection and RNA extraction. Among the 41 clinical samples obtained from eight patients with laboratory-confirmed COVID-19, four (9.8%) tested positive in commercial RT-qPCR assays. Two of these samples (#P1-S_20200214 and #P2-P_20200406) showed weak positive signals with higher Ct values in the E and RdRP genes and not all viral genes were detected (Table 1). According to the manufacturer’s instructions, ‘#P2-P_20200406’ may be more appropriately classified as a negative sample for SARS-CoV-2 since only the E gene was detected (Ct = 37) while the other two genes were negative. We formulated some hypotheses to explain the positivity of only the E gene, such as the sample being obtained during the patient’s remission phase of infection and/or having a very low viral load.

### 3.3. Condensed Extraction of Total RNA Is Useful for Detection by In-House RT-PCR without Viral Culture

The conventional procedure for RNA extraction is typically optimized with 140 µL samples. In this study, we employed a condensed method to circumvent the need for virus culture and BSL-3 facilities. To amplify the entire SARS-CoV-2 genome, we divided it into five fragments, each approximately 6 kb in length (Figure S1). The results showed that clinical specimens with lower Ct values were more amenable to amplification by in-house RT-PCR, indicating that gene amplification efficiency was influenced by the Ct value (Ct < 38) (Table 1 and Figure S1). Notably, the in-house RT-PCR results provided supportive information for the commercial RT-qPCR. Despite the low viral RNA concentration in plasma specimens, sample #P2-P_20200406 (labeled as #P2-plasma-4) was identified as SARS-CoV-2-positive and confirmed through our experimental analysis (Figure S1).

To assess the cross-reactivity among related coronaviruses, including MERS-CoV, SARS-CoV, and SARS-CoV-2, we utilized the complete genomic sequences and compared the nucleotide locations of 30 primer pairs. The reference sequences for the related coronaviruses are as follows: MERS-CoV (Accession no: NC_019843/Version: NC_019843.3/length: 30,119 bp), SARS-CoV (NC_004718/NC_004718.3/29,751 bp), and SARS-CoV-2 (NC_045512/NC_045512.2/29,903 bp). Using ClustalW for multiple sequence alignments, three sequences were divided into two groups. SARS-CoV and SARS-CoV-2 exhibited a higher level of similarity (80%), prompting us to analyze the cross-reactivity between these two viruses (Table S2). By using the nucleotide sequence of SARS-CoV-2 as a template, we examined the corresponding positions in the SARS-CoV sequence. While there is a possibility that a limited number of sequencing primers could detect the sequence of SARS-CoV, we conducted direct sequencing using the 6 kb products to prevent any potential misidentification (Table S2 and Figure S1).

### 3.4. Codon and Amino Acid Usage Patterns

The spike protein of SARS-CoV-2 is known to interact with the host cell receptor angiotensin-converting enzyme 2 (ACE2) for virus entry [23]. In this study, we compared the amino acid usage patterns in the spike protein between globally recognized variant strains and early cases of SARS-CoV-2 infection in Taiwan (Figure S2). We identified non-synonymous substitutions in the nucleotide sequences of two early COVID-19-confirmed patients, located in the ORF1ab/non-structural protein 3 (Nsp3) (at position 6312, C to A) and a silent mutation in the spike gene (position 23929, C to T). Additionally, the sequence of #P7 showed a synonymous substitution in ORF1ab/Nsp3 (position 6061, T to G). To (1) understand whether the spike protein sequences of the two early infected individuals (TWN/NYCU-P7 & TWN/NYCU-P10) in Taiwan, as revealed in this article, are more similar to NC045512.2 or have undergone an evolution, and (2) demonstrate that viral strains evolve and change over time. Therefore, we used the 1273 amino acids of the spike protein from NC045512.2 as a reference for comparing the variations in commonly seen viral strains internationally. Supplemental Figure S2 illustrates the distribution of non-synonymous substitutions within the spike protein, with a concentration of mutations observed in its S1 subunit (residues 14–685). The amino acid sequences of the two early infections in this study showed higher similarity to the NC045512.2 compared to other consensus sequences. Moreover, certain discordant motifs were observed within the same consensus groups. Taking clade B for example, it has undergone variations over time, with a common deletion occurring at position 49 (H49-). Additionally, inconsistencies are observed at positions 70, 74, 797, and 884. The positions that can be used to identify different viral variants include Alpha (P681H, T716I, S982A, D1118H), Beta (D80A, D215G, A701V), and Gamma (T20N, P26S, D138Y, K417T, H655Y, T1027I, V1176F). Notably, the variants Alpha, Beta (B.1.351/B.1.351.2/B1.351.3), and Gamma (P.1) exhibited common mutations, including N501Y and D614G (Figure S2).

### 3.5. Phylogenetic Trees

**Figure 2:**
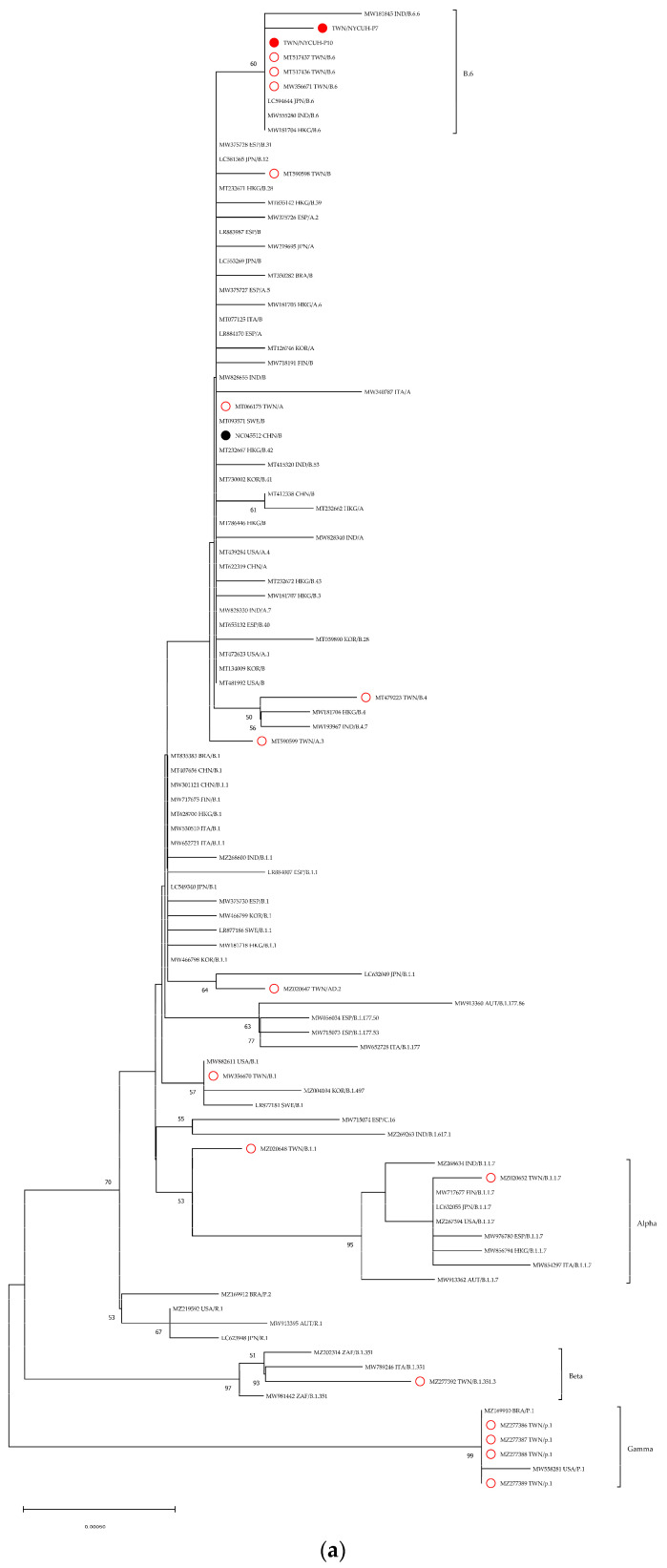

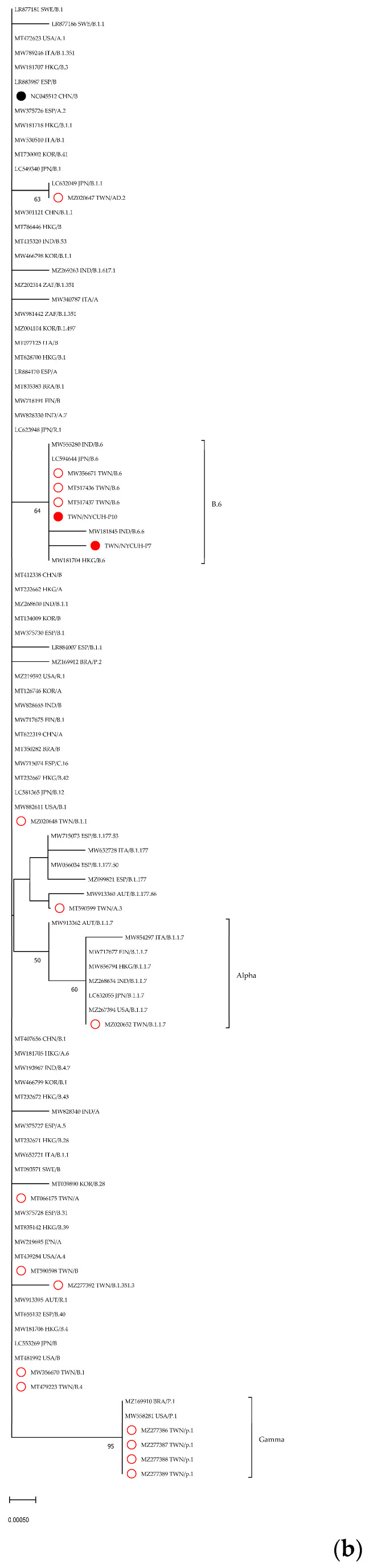
Phylogenetic analysis of SARS-CoV-2 strains that circulated throughout the world. There were 102 nucleotide sequences in the final dataset. Different lengths were used to build unrooted phylogenetic trees. Neighbor-joining trees based on the proposed method (**a**) assembled sequence of all variant regions (v1–v5, 6274 bp) and (**b**) partial sequence of Nsp3/PLpro (v3, 1401 bp) aligned by the current study.

Recombination events can occur in SARS-CoV-2 [24]. Our study identified five regions in the entire genome sequence that exhibited poor similarity (<95%). These regions were selected as potential candidates for further analysis (Figure S3). We assembled these regions (v1–v5, approximately 6.5 Kb) and prepared a shorter sequence consisting of only v3 (~1.4 Kb) for comparison. To demonstrate the efficiency of assembling sequences from specific regions of the genome, we compared datasets of different lengths for molecular evolutionary genetic analysis (Figure 2, Figure S4 and Figure S5). Two sequences from early Taiwanese SARS-CoV-2 infections in our study (#P7 and #P10, marked with red spots) clustered with lineage B.6. Furthermore, other early Taiwanese sequences were also included in the phylogenetic trees and marked with red circles. When comparing the robustness of the trees generated by our proposed method versus the conventional method, we focused on the bootstrap values within the B.6 lineage cluster. In the neighbor-joining (NJ) trees, the bootstrap values for the nodes were 72% for the conventional method, while the proposed methods achieved 60% and 64% (Figure S4 and Figure 2). Similar outcomes were observed in the maximum likelihood (ML) trees generated by these methods (Figure S5). Overall, when incorporating all variant regions (v1–v5), the evolutionary analysis tends to produce results that closely align with the branching patterns observed in the analysis of the full-length sequences. This approach not only enables effective differentiation of specific variants (such as Alpha, Beta, and Gamma) but also reveals a distinct cluster formed by the B.6 lineage.

## 4. Discussion

SARS-CoV-2 has spread to over 200 countries, and serosurveillance studies reveal differences in virus exposure and antibody-mediated immunity based on demographics and healthcare settings [25]. Serological diagnosis is particularly important for asymptomatic cases and patients with mild-to-moderate disease. Understanding the drivers of SARS-CoV-2 exposure and quantifying population immunity are crucial for future epidemic preparedness.

The timeline of COVID-19 diagnostic markers has been established based on available evidence [26,27]. During this pandemic, hematologic laboratories play a vital role in patient screening, diagnosis, and prognosis. Sample-pooling approaches facilitate mass screening in the early stages of outbreaks, and commercial RT-qPCR assays have been developed for COVID-19 confirmation. Serologic tests for SARS-CoV-2 are becoming widely available to assess viral exposure and collect population-based blood samples for validation studies [28]. However, the documentation of false negative results, methodological comparisons among RT-qPCR assays/platforms, and the validity of rapid diagnostic tests (RDTs) are often inadequate [27].

The timeline of antibody-mediated immune responses involving IgM, IgA, IgG, and IgE follows the decrease in viral RNA [26]. Obesity may impact the efficacy of SARS-CoV-2 vaccines, with diminished protective effects observed in obese individuals compared to those with a healthy weight [29]. Clinical inquiries and observational database assessments in Taiwan indicate higher morbidity and mortality in obese individuals with COVID-19. Although SARS-CoV-2 can be detected by RT-qPCR in various clinical specimens, only a small percentage of blood samples tested positive [30,31]. This suggests that transmission of the virus primarily occurs through respiratory and other routes. Due to the timing of sample collection, not all throat swabs from patients were successfully amplified by RT-qPCR. In this study, we employed several serological assays based on indirect ELISA to validate the antibody response at various time points. Except for patient 1 (#P1), all other patients showed SARS-CoV-2 antibody response in the ELISA assays detecting virus-specific IgG antibodies. Our data support the combined use of nucleic acid amplification and serological tests for maximum case detection.

Genomic analysis of SARS-CoV-2 is crucial for understanding its origin and structure. The B.6 lineage, which originated from the Asia Pacific [32], accounts for more than 95% of global isolates and was predominant during the early pandemic. Positive selection drives the pathogenicity of SARS-CoV-2 [33], leading to viral divergence and the emergence of multiple variants. Most substitutions occur in the ORF1ab gene, particularly in the segment encoding Nsp3, which includes the papain-like protease (PLpro) [34]. PLpro plays a role in polyprotein cleavage [35] and is a potential target for drug discovery [36]. Although specific targeted therapies are currently lacking, antiviral drugs such as Lopinavir/Ritonavir have been tested against SARS-CoV-2, similar to other viral infections [37]. Further genomic analysis focused on Nsp3/PLpro may provide insights into molecular adaptation, recombination events, and drug resistance monitoring.

To address concerns about variant strains, we confirmed the specificity of our homemade primer pairs through comparative sequence analysis. A bootstrap value of 70% is often considered a reliable cluster cutoff, and our findings suggest that the tree generated by the proposed method is more reliable, less time-consuming, and more efficient than the conventional method. This article presents the first validation and use of in-house RT-PCR for amplifying the full SARS-CoV-2 sequence, providing valuable information for countries and regions.

## 5. Conclusions

The study’s impacts are as follows: (a) The study emphasizes the critical role of RNA extraction concentration in detecting samples with lower Ct values. (b) All experiments were conducted in BSL-2 facilities without the need for viral culture. (c) The study demonstrates that the methods used can be performed using conventional techniques, eliminating the requirement for expensive facilities. (d) The selection of high-variable regions for phylogenetic analysis is shown to be more efficient. (e) Nsp3/PLpro analysis provides valuable insights for monitoring drug resistance.

## Figures and Tables

**Table 1:** Details of clinical specimen.

Specimens ^a^	Total RNA (ng/µL)	RT-PCR Performed	The C_t_ Value of RT-qPCR ^b^			
			E	RdRP-R2	N	RNase P
#P1-S_20200214	122.1	V	33.48 (+)	33.97 (+)	− (−)	27.27 (+)
#P1-P_20200207	44.4		− (−)	− (−)	− (−)	32.39 (+)
#P1-P_20200211	36.5		− (−)	− (−)	− (−)	31.82 (+)
#P2-P_202003xx	48.6		− (−)	− (−)	− (−)	29.53 (+)
#P2-P_20200325	134.3		− (−)	− (−)	− (−)	26.47 (+)
#P2-P_20200330	93.8		− (−)	− (−)	− (−)	31.71 (+)
#P2-P_20200402	149.6		− (−)	− (−)	− (−)	31.29 (+)
#P2-P_20200406	68.9	V	37.05 (+)	− (−)	− (−)	28.06 (+)
#P2-P_20200410	82.0		− (−)	− (−)	− (−)	27.51 (+)
#P7-S_20200423	113.7	V	20.93 (+)	22.32 (+)	25.58 (+)	24.55 (+)
#P7-P_20200423	42.6		− (−)	− (−)	− (−)	32.08 (+)
#P7-P_20200427	110.4		− (−)	− (−)	− (−)	28.73 (+)
#P7-P_20200430	70.0		− (−)	− (−)	− (−)	31.19 (+)
#P7-P_20200504	53.2		− (−)	− (−)	− (−)	32.42 (+)
#P7-P_20200507	74.4		− (−)	− (−)	− (−)	30.28 (+)
#P7-P_20200511	76.9		− (−)	− (−)	− (−)	33.15 (+)
#P8-S_20200427	140.1		− (−)	− (−)	− (−)	25.75 (+)
#P8-P_20200427	120.0		− (−)	− (−)	− (−)	27.44 (+)
#P8-P_20200430	87.6		− (−)	− (−)	− (−)	30.82 (+)
#P8-P_20200504	117.5		− (−)	− (−)	− (−)	31.36 (+)
#P8-P_20200507	125.6		− (−)	− (−)	− (−)	28.91 (+)
#P8-P_20200511	97.4		− (−)	− (−)	− (−)	31.20 (+)
#P9-S_20200427	112.3		− (−)	− (−)	− (−)	27.74 (+)
#P9-P_20200427	125.4		− (−)	− (−)	− (−)	− (−)
#P9-P_20200430	60.6		− (−)	− (−)	− (−)	30.10 (+)
#P9-P_20200504	62.9		− (−)	− (−)	− (−)	30.95 (+)
#P9-P_20200507	37.4		− (−)	− (−)	− (−)	31.15 (+)
#P10-S_20200427	150.6	V	17.50 (+)	22.59 (+)	19.14 (+)	23.89 (+)
#P10-P_20200427	51.3		− (−)	− (−)	− (−)	− (−)
#P10-P_20200430	35.2		− (−)	− (−)	− (−)	31.44 (+)
#P10-P_20200504	42.8		− (−)	− (−)	− (−)	32.62 (+)
#P10-P_20200507	22.7		− (−)	− (−)	− (−)	32.40 (+)
#P10-P_20200511	34.4		− (−)	− (−)	− (−)	33.00 (+)
#P11-S_20200427	140.0		− (−)	− (−)	− (−)	27.33 (+)
#P11-P_20200427	72.1		− (−)	− (−)	− (−)	29.43 (+)
#P11-P_20200430	71.8		− (−)	− (−)	− (−)	30.82 (+)
#P11-P_20200504	25.9		− (−)	− (−)	− (−)	33.52 (+)
#P12-S_20200427	140.9		− (−)	− (−)	− (−)	24.14 (+)
#P12-P_20200427	33.5		− (−)	− (−)	− (−)	30.22 (+)
#P12-P_20200430	42.3		− (−)	− (−)	− (−)	28.93 (+)
#P12-P_20200511	108.9		− (−)	− (−)	− (−)	31.19 (+)

^a^ Clinical specimen S means throat swab and P means plasma. ^b^ Target genes (E, N, RdRP, and RNase P) were detected by commercial RT-qPCR assays, combined results of the C_t_ value as shown in the brackets (+ means positive reaction and − means negative reaction).

## Data Availability

The data presented in this study are available in Section 2.2. Part of this article was presented at the 9th First Member Conference and Academic Symposium in Taiwan in 2022 (poster, 111-P01).

## Supplementary Materials

The following supporting information can be downloaded at: https://www.mdpi.com/article/10.3390/v15071562/s1. Figure S1: The results of the PCR products are loaded directly onto 1% agarose TBE gels after in-house RT-PCR; Figure S2: comparison of amino acid usage patterns in spike protein between commonly known strains and early cases infected with SARS-CoV-2 from Taiwan; Figure S3: using SimPlot (version 3.5.1) to determine the characteristics of the virus strain, putative recombinants, and their similarity; Figure S4: phylogenetic analysis of SARS-CoV-2 strains that circulated throughout the world (near full-length, NJ tree); Figure S5: phylogenetic analysis of SARS-CoV-2 strains that circulated throughout the world (v1–v5 & v3 only, ML trees); Table S1: primer pairs and RT-PCR conditions; Table S2: using the complete genomic sequences, assess the cross-reactivity between SARS-CoV and SARS-CoV-2 by comparing the nucleotide locations of 30 primer pairs.
