# Supplementary material for: A Comprehensive Phylogenetic Analysis of SARS-CoV-2: Utilizing a Novel and Convenient In-House RT-PCR Method for Characterization without Virus Culture and BSL-3 Facilities
Source: Viruses. 2023 Jul 16;15(7):1562. doi: 10.3390/v15071562 (PMC10383548; doi:10.3390/v15071562)
Supplement: Supplementary file 1 [file viruses-15-01562-s001.zip › viruses-2455028 supplementary.pdf]

Table S1. Primer pairs and RT-PCR conditions

Name	Location I [a]	Sequence	Nonstructural proteins [b]	Coding region
nCoV-1F	1-24	5′- ATTAAAGGTTTATACCTTCCCAGG -3′	nsp1	ORF1a
nCoV-1R	1098-1076	5′- TTTAAGGGAAATACAAAATTTGG -3′	nsp1 & nsp2	
nCoV-2F	1004-1026	5′- AAGAGCTATGAATTGCAGACACC -3′	v1 nsp2	
nCoV-2R	2101-2081	5′- CCACTGCGAAGTCAACTGAAC -3′	nsp2	
nCoV-3F	1997-2017	5′- CTGAGACTCATTGATGCTATG -3′	nsp2	
nCoV-3R	3124-3101	5′- ATCTTCAGTACCATACTCATATTG -3′	nsp2 & nsp3	
nCoV-4F	3040-3059	5′- CCCTCCAGATGAGGATGAAG -3′	nsp3	
nCoV-4R	4132-4109	5′- ATCACCCACTATATATGGAGCATC -3′	v2 nsp3	
nCoV-5F	4036-4059	5′- TGACATTAATGGCAATCTTCATCC -3′	nsp3	
nCoV-5R	5102-5079	5′- GTGAATTATGAGGTTTTATTTTAG -3′	nsp3	
nCoV-6F	4979-5002	5′- ACAACAGTAGACAACATTAACCTC -3′	nsp3	
nCoV-6R	6100-6078	5′- AGTTAACTGGTTTAAATCATCAG -3′	nsp3	
nCoV-7F	6017-6040	5′- CAACCATATCCAAACGCAAGCTTC -3′	v3 nsp3	
nCoV-7R	7104-7081	5′- GTTGCAATAGTGACATTAGTAGAG -3′	nsp3	
nCoV-8F	6968-6990	5′- CTATTAAGTGTTTGCCTAGGTTC -3′	nsp3	
nCoV-8R	8090-8067	5′- GTTTTTCCATTGGTACGTTAAAAG -3′	nsp3	
nCoV-9F	7973-7996	5′- CTGTTACTAGATCAGGCATTAGTG -3′	v4 nsp3	
nCoV-9R	9091-9067	5′- AGAACCTTCTAGTACATTGGTATC -3′	nsp3 & nsp4	
nCoV-10F	8974-8997	5′- AGAGTACACTGACTTTGCAACATC -3′	nsp4	
nCoV-10R	10135-10112	5′- AAGTGTAGTTGTACCACAAGTTAC -3′	nsp4 & nsp5	
nCoV-11F	10002-10025	5′- CTGATGTTCTTTACCAACCACCAC -3′	nsp5	
nCoV-11R	11069-11048	5′- AAGACCATTGAGTACTCTGGAC -3′	nsp5 & nsp6	
nCoV-12F	10973-10996	5′- AGTGCAGTGAAAAGAACAATCAAG -3′	nsp6	
nCoV-12R	12110-12089	5′- TAAACTCTGAGGCTATAGCTTG -3′	nsp6, nsp7, & nsp8	
nCoV-13F	11998-12019	5′- GGTTTCACTACTTTCTGTTTTG -3′	nsp8	
nCoV-13R	13123-13101	5′- ACTAGCTAGATAATCTTTGTAAG -3′	nsp8, nsp9, & nsp10	
nCoV-14F	12982-13004	5′- AGGTATGGTACTTGGTAGTTTAG -3′	nsp10	ORF1a & ORF1b
nCoV-14R	14108-14085	5′- ATGAAATCACCGAAATCATACCAG -3′	nsp11, nsp12	
nCoV-15F	13960-13981	5′- GTATACGCCAACTTAGGTGAAC -3′	nsp12	ORF1b
nCoV-15R	15135-15113	5′- AGTACTACAGATAGAGACACCAG -3′	nsp12	
nCoV-16F	15031-15053	5′- ACAAAACGTAATGTCATCCCTAC -3′	nsp12	
nCoV-16R	16086-16065	5′- ATGAAAGACATCAGCATACTCC -3′	nsp13	
nCoV-17F	15934-15955	5′- CCAGATCCATCAAGAATCCTAG -3′	nsp12 & nsp13	
nCoV-17R	17125-17104	5′- GAGCTAGGCCAATAGCAAAATG -3′	nsp13	ORF1b
nCoV-18F	17013-17035	5′- AGATGAGTTTTCTAGCAATGTTG -3′	nsp13	

nCoV-18R	18120-18101	5′- GAGGTGTGTAGGTGCCTGTG -3′	nsp14	
nCoV-19F	18019-18040	5′- AGGAATGTGGCAACTTTACAAG -3′	nsp14	
nCoV-19R	19070-19049	5′- GCTTGAGGTACACACTTAATAG -3′	nsp14	
nCoV-20F	18928-18951	5′- CCTATAATTGGTGATGAACTGAAG -3′	nsp14 & nsp15	
nCoV-20R	20119-20099	5′- ATGTGACTCCATTAAGACTAG -3′	nsp15	
nCoV-21F	19994-20016	5′- GTAGAGTTGATGGTCAAGTAGAC -3′	nsp15 & nsp16	
nCoV-21R	21077-21055	5′- GTAACATTTTTAGTCTTAGGGTC -3′	nsp16	
nCoV-22F	20962-20984	5′- GACTTTGTCTCTGATGCAGATTC -3′	nsp16	ORF1b & S
nCoV-22R	22103-22082	5′- CTTCAAGGTCCATAAGAAAAGG -3′	nsp16	
nCoV-23F	22007-22030	5′- AACAAAAGTTGGATGGAAAGTGAG -3′	v5	S
nCoV-23R	23131-23111	5′- AGTTGCTGGTGCATGTAGAAG -3′		
nCoV-24F	23040-23061	5′- AATCATATGGTTTCCAACCCAC -3′		
nCoV-24R	24116-24096	5′- CACAAATGAGGTCTCTAGCAG -3′		
nCoV-25F	24009-24031	5′- CATTTATTGAAGATCTACTTTTC -3′		
nCoV-25R	25117-25096	5′- GCGGTCAATTTCTTTTTGAATG -3′		
nCoV-26F	24988-25010	5′- ACCTGAATTAGACTCATTCAAGG -3′		S & ORF3a
nCoV-26R	26163-26143	5′- ATTAACAACTCCGGATGAACC -3′		
nCoV-27F	26041-26063	5′- ACTCAATTGAGTACAGACACTGG -3′		ORF3a, E, & M
nCoV-27R	27126-27106	5′- CAATCCTGTAGCGACTGTATG -3′		
nCoV-28F	27024-27046	5′- ATCACTGTTGCTACATCACGAAC -3′		M, ORF6, ORF7a, ORF7b, & ORF 8
nCoV-28R	28143-28122	5′- AACAGGAAACTGTATAATTACC -3′		
nCoV-29F	28038-28060	5′- GTAGGAGCTAGAAAATCAGCACC -3′		ORF8 & N
nCoV-29R	29116-29096	5′- TTGTTCTGGACCACGTCTGCC -3′		
nCoV-30F	28937-28958	5′- CTGCTGCTTGACAGATTGAACC -3′		N & ORF10
nCoV-30R	29861-29837	5′- CTAAGAAGCTATTAAAATCACATGG -3′		

[a] Location from the start of NC045512 nucleotide sequence (Wuhan-Hu-1, coronavirus 2 isolate of severe acute respiratory syndrome, complete genome) 1 → 29870.

[b] Chan *et al.* provided predictions for the amino acid positions of nonstructural proteins as follows: nsp1 (M1 - G180), nsp2 (A181 - G818), nsp3 (A819 - G2763), nsp4 (K2764 - Q3263), nsp5 (S3264 - Q3569), nsp6 (S3570 - Q3859), nsp7 (S3860 - Q3942), nsp8 (A3943 - Q4140), nsp9 (N4141 - Q4253), nsp10 (A4254 - Q4392), nsp11 (S4393 - V4405), nsp12 (S4393 - Q5324), nsp13 (A5325 - Q5925), nsp14 (A5926 - Q6452), nsp15 (S6453 - Q6798), and nsp16 (S6799 - N7096).

RNA extraction:

Regarding the condensed extraction of RNA, the procedures are as follows:

1. Pipet 560 µl prepared Buffer AVL containing carrier RNA into a 1.5 ml microcentrifuge tube.
2. Add 140 µl plasma to the Buffer AVL–carrier RNA in the microcentrifuge tube. Mix by pulse-vortexing for 15 s. Incubate at room temperature for 10 min. Briefly centrifuge the tube to remove drops from the inside of the lid.

3. Add 560 µl ethanol (96–100%) to the sample, and mix by pulse-vortexing for 15 s. After mixing, briefly centrifuge the tube to remove drops from inside the lid. (steps 1 - 3, total volume: 1260 µl).

Note: In this study, we utilized a double volume of plasma (2 * 140 µl) for each clinical sample. This means that each sample needed to be divided into two tubes (2 * 1260 µl) and subsequently pooled into a single extraction column.

4. Carefully apply 630 µl of the lysate from step 3 into the QIAamp Mini column (in a 2 ml collection tube) without wetting the rim. Avoid touching the QIAamp Mini column membrane with the pipette tip. Close the cap, and centrifuge at 6000 x g (8000 rpm) for 1 min. Place the QIAamp Mini column into a clean 2 ml collection tube, and discard the tube containing the filtrate.
5. Carefully open the QIAamp Mini column, and repeat step 4 until all of the lysate (2* 1260 µl) has been drawn through the QIAamp Mini column.
6. Carefully open the QIAamp Mini column, and add 500 µl Buffer AW1 to the QIAamp Mini column without wetting the rim (in a 2 ml collection tube). Close the cap, and centrifuge at 6000 x g (8000 rpm) for 1 min. Place the QIAamp Mini column into a clean 2 ml collection tube, and discard the tube containing the filtrate.
7. Carefully open the QIAamp Mini column, and add 500 µl Buffer AW2 to the QIAamp Mini column without wetting the rim (in a 2 ml collection tube). Close the cap, and centrifuge at 20000 x g (14000 rpm) for 3 min. Place the QIAamp Mini column into a clean 2 ml collection tube, and discard the tube containing the filtrate.
8. Place the QIAamp Mini column in a new 2 ml collection tube, and centrifuge at 20000 x g (14000 rpm) for 1 min.
9. Place the QIAamp Mini column in a clean 1.5 ml microcentrifuge tube. Discard the old collection tube containing the filtrate. Carefully open the QIAamp Mini column and add 60 µl Buffer AVE equilibrated to room temperature. Close the cap, and incubate at room temperature for 15 min.
10. Centrifuge at 6000 x g (8000 rpm) for 2 min, and discard the QIAamp Mini column. Finally, the concentrated Viral RNA extraction was stored at -80°C.

RT-PCR conditions:

1. Real-time RT-PCR (Roche Lightcycler® multiplex RNA virus master, Cat No: 06754155001)

Briefly, the 20 µL RT-qPCR mixture contained the RT enzyme solution, the RT-qPCR reaction mix, 10 µM each of the forward and reverse primer, 5 µM probe, 5 µL extracted RNA or water for the controls without template. The samples were also processed and analyzed using the Roche Lightcycler 480 under the following conditions: 10 min at 50 °C and 30 s at 95 °C, followed by 45 cycles of 15 s at 95 °C and 53 s at 60 °C. The positive detection of COVID-19 was considered by the Taiwan CDC protocol when a sample was positive for the E, RdRp, and N genes. The samples were considered negative for COVID-19 if they were negative for the E and RdRp genes, or negative for the RdRp gene but positive for the E gene.

2. In-house RT-PCR

2.1. cDNA synthesis (Thermo Scientific™ RevertAid RT Reverse Transcription Kit, Product code: 15255146)

Component	Volume

Step	ºC	Time	Cycles

Random primer	1 ul
Total RNA (~500 ng)	-
Nuclease-free water	to 12 ul
5X Reaction Buffer	4 ul
10 mM dNTP Mix	2 ul
RNase Inhibitor	1 ul
RevertAid RT	1 ul
Total	20 ul

Optional	65	5 min	1
	25	5 min	1
RT reaction	42	60 min	1
Extension	70	5 min	1
Hold	4	∞	1

2.2. 1st PCR (Invitrogen™ Platinum™ SuperFi™ II DNA Polymerase, Cat No: 12361010)

Component	Volume
nCoV-1F/-11F (10 uM)	1 ul
nCoV-20R/-30R(10 uM)	1 ul
cDNA	1 ul
5X SuperFi™ II Buffer	10 ul
10 mM dNTPs	1 ul
Platinum™ SuperFi™ II DNA polymerase	1 ul
Water	35 ul
Total	50 ul

Step	ºC	Time	Cycles
Initial Denaturation	98	30 s	1
Denaturation	98	10 s	
Annealing	60	10 s	35
Extension	72	10 min	
Final extension	72	5 min	1
Hold	4	∞	

2.3. Nested PCR (ALLin™ HiFi DNA Polymerase, Cat No: HLE0201)

Component	Volume
5x buffer	6 ul
nCoV-xF (10 uM)	1.5 ul
nCoV-xR (10 uM)	1.5 ul
PCR product	1 ul
HS HiFi DNA polymerase	0.3 ul
Nuclease-free water	19.7 ul
Total	30 ul

Step	ºC	Time	Cycles
Initial Denaturation	95	1 min	1
Denaturation	95	15 s	
Annealing (Tm)	60	15 s	35
Extension	72	3 min	
Final extension	72	10 min	1
Hold	4	∞	

Table S2. Using the complete genomic sequences, assess the cross-reactivity between SARS-CoV and SARS-CoV-2 by comparing the nucleotide locations of 30 primer pairs

	SARS-CoV-2 (Accession no. NC_045512)	SARS-CoV (Accession no. NC_004718) [a]
nCoV-1F	5′- ATTAAAGGTTTATACCTTCCCAGG -3′	5′- ATATTAGGTTTTTACCTACCCAGG -3′
nCoV-1R	5′- TTTAAGGGAAATACAAAATTTGG -3′	5′- TTAAGAGGAAACACAAACTTTGG -3′
nCoV-2F	5′- AAGAGCTATGAATTGCAGACACC -3′	5′- AAGAGCTACGAGCACCAGACACC -3′
nCoV-2R	5′- CCACTGCGAAGTCAACTGAAC -3′	5′- CCACTGAGAAGTCTGTTGTAC -3′
nCoV-3F	5′- CTGAGACTCATTGATGCTATG -3′	5′- TTACGTCTTGTCGACGCCATG -3′

nCoV-3R	5′- ATCTTCAGTACCATACTCATATTG -3′	5′- ATCCTCTGTACCGTACTCATGTTC -3′
nCoV-4F	5′- CCCTCCAGATGAGGATGAAG -3′	5′- CCCTCCAGATGAGGAAGAAG -3′
nCoV-4R	5′- ATCACCCACTATATATGGAGCATC -3′	5′- ATCACCTACCATGTAAGGTGCATC -3′
nCoV-5F	5′- TGACATTAATGGCAATCTTCATCC -3′	5′- TGATATCAATGGTAAGCTTTACCA -3′
nCoV-5R	5′- GTGAATTATGAGGTTTTATTTTAG -3′	5′- GATTTACATGAGGTTTAATTTTTG -3′
nCoV-6F	5′- ACAACAGTAGACAACATTAACCTC -3′	5′- ACAACTGTGGACAACACTAATCTC -3′
nCoV-6R	5′- AGTTAACTGGTTTAAATCATCAG -3′	5′- TGTCATTTGATTTAAATCATCAG -3′
nCoV-7F	5′- CAACCATATCCAAACGCAAGCTTC -3′	5′- CAACCATTACCAAATGCGAGTTTT -3′
nCoV-7R	5′- GTTGCAATAGTGACATTAGTAGAG -3′	5′- TCCATAGTAGTAACGTTAGACGAA -3′
nCoV-8F	5′- CTATTAAGTGTTTGCCTAGGTTC -3′	5′- TTGTTAAGTATTTGCTTAGGTTC -3′
nCoV-8R	5′- GTTTTTCCATTGGTACGTTAAAAG -3′	5′- GTTTTTCCATAGGAACACTAAAAG -3′
nCoV-9F	5′- CTGTTACTAGATCAGGCATTAGTG -3′	5′- CTGTTGCTTGACCAAGCTCTTGTA -3′
nCoV-9R	5′- AGAACCTTCTAGTACATTGGTATC -3′	5′- AGAACCCTCTAGCAAATTAGTGTC -3′
nCoV-10F	5′- AGAGTACACTGACTTTGCAACATC -3′	5′- TGAGTATAGTGATTTTGCTACCTC -3′
nCoV-10R	5′- AAGTGTAGTTGTACCACAAGTTAC -3′	5′- AAGAGTTGTAGTTCCACAGGTTAC -3′
nCoV-11F	5′- CTGATGTTCTTTACCAACCACCAC -3′	5′- CTGATGTTCTCTACCAACCACCAC -3′
nCoV-11R	5′- AAGACCATTGAGTACTCTGGAC -3′	5′- GTGACCACTGTGTACTTTGAAC -3′
nCoV-12F	5′- AGTGCAGTGAAAAGAACAATCAAG -3′	5′- GGTAAGTTCAAGAAAATTGTTAAG -3′
nCoV-12R	5′- TAAACTCTGAGGCTATAGCTTG -3′	5′- TAAATTCTGAAGCAATAGCCTG -3′
nCoV-13F	5′- GGTTTCACTACTTTCTGTTTTG -3′	5′- GGTTTCTCTTTTGTCTGTTTTG -3′
nCoV-13R	5′- ACTAGCTAGATAATCTTTGTAAG -3′	5′- ACTTGCTAGGTAATCCTTATATG -3′
nCoV-14F	5′- AGGTATGGTACTTGGTAGTTTAG -3′	5′- AGGTATGGTGCTGGGCAGTTTAG -3′
nCoV-14R	5′- ATGAAATCACCGAAATCATACCAG -3′	5′- ACGAAATCACCGAAATCGTACCAG -3′
nCoV-15F	5′- GTATACGCCAACTTAGGTGAAC -3′	5′- GTATATGCTAACTTAGGTGAGC -3′
nCoV-15R	5′- AGTACTACAGATAGAGACACCAG -3′	5′- AGTACTACAGATAGAGACACCAG -3′
nCoV-16F	5′- ACAAAACGTAATGTCATCCCTAC -3	5′- ACTAAGCGTAATGTCATCCCTAC -3′
nCoV-16R	5′- ATGAAAGACATCAGCATACTCC -3′	5′- GTGAAAGACATCAGCATACTCC -3′
nCoV-17F	5′- CCAGATCCATCAAGAATCCTAG -3′	5′- CCAGATCCATCAAGAATATTAG -3′
nCoV-17R	5′- GAGCTAGGCCAATAGCAAAATG -3′	5′- GAGCAAGTCCGATGGCAAAATG -3′
nCoV-18F	5′- AGATGAGTTTTCTAGCAATGTTG -3′	5′- AGATGAGTTTTCTAGCAATGTTG -3′
nCoV-18R	5′- GAGGTGTGTAGGTGCCTGTG -3′	5′- GAGGTGTGTAGGTGCCTGTG -3′
nCoV-19F	5′- AGGAATGTGGCAACTTTACAAG -3′	5′- CGCAATGTGGCTACATTACAAG -3′
nCoV-19R	5′- GCTTGAGGTACACACTTAATAG -3′	5′- GCCTGAGGCACACACTTGATAG -3′
nCoV-20F	5′- CCTATAATTGGTGATGAACTGAAG -3′	5′- CCTATTATAGGAGATGAACTGAGG -3′
nCoV-20R	5′- ATGTGACTCCATTAAGACTAG -3′	5′- ATGTGACTCCATTGACGCTAG -3′
nCoV-21F	5′- GTAGAGTTGATGGTCAAGTAGAC -3′	5′- GTAGAGTGGAAGGACAGGTAGAC -3′
nCoV-21R	5′- GTAACATTTTTAGTCTTAGGGTC -3′	5′- GTCACATGTTTGGTCCTAGGGTC -3′
nCoV-22F	5′- GACTTTGTCTCTGATGCAGATTC -3′	5′- GACTTCGTCTCCGACGCAGATTC -3′
nCoV-22R	5′- CTTCAAGGTCCATAAGAAAAGG -3′	5′- CTGAAACATCAAGCGAAAAGGC -3′
nCoV-23F	5′- AACAAAAGTTGGATGGAAAGTGAG -3′	5′- A-CACAGAC---ACATACTATGAT -3′
nCoV-23R	5′- AGTTGCTGGTGCATGTAGAAG -3′	5′- CGTGGCCGGTGCATTTAAAAG -3′
nCoV-24F	5′- AATCATATGGTTTCCAACCCAC -3′	5′- ATGATTATGGTTTTTACACCAC -3′

nCoV-24R	5′- CACAAATGAGGTCTCTAGCAG -3′	5′- CACAAATGAGATCTCTAGCAT -3′
nCoV-25F	5′- CATTTATTGAAGATCTACTTTTC -3′	5′- CTTTTATTGAGGACTTGCTCTTT -3′
nCoV-25R	5′- GCGGTCAATTTCTTTTTGAATG -3′	5′- GCGGTCAATTTCTTTTTGAATG -3′
nCoV-26F	5′- ACCTGAATTAGACTCATTCAAGG -3′	5′- ACCTGAGCTTGACTCATTCAAAG -3′
nCoV-26R	5′- ATTAACAACTCCGGATGAACC -3′	5′- ATTAGCAACTCCTGAAGAGCC -3′
nCoV-27F	5′- ACTCAATTGAGTACAGACACTGG -3′	5′- ACACAAATTACTACAGACACTGG -3′
nCoV-27R	5′- CAATCCTGTAGCGACTGTATG -3′	5′- CAATACGGTAGCGGTTGTATG -3′
nCoV-28F	5′- ATCACTGTTGCTACATCACGAAC -3′	5′- ATCACTGTGGCTACATCACGAAC -3′
nCoV-28R	5′- AACAGGAAACTGTATAATTACC -3′	5′- GGTGTGCATGTTTG-AACCATA -3′
nCoV-29F	5′- GTAGGAGCTAGAAAATCAGCACC -3′	5′- CTAGGGGTAATACTTATAGCACT -3′
nCoV-29R	5′- TTGTTCTGGACCACGTCTGCC -3′	5′- TTGTTCTGGACCACGTCTCCC -3′
nCoV-30F	5′- CTGCTGCTTGACAGATTGAACC -3′	5′- TTGCTGCTAGACAGATTGAACC -3′
nCoV-30R	5′- CTAAGAAGCTATTAAAATCACATGG -3′	5′- CTAAGAAGCTATTAAAATCACATGG -3′

[a] Using the nucleotide sequence of SARS-CoV-2 as a template, display the sequence of SARS-CoV at the corresponding positions.

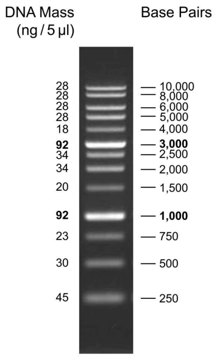

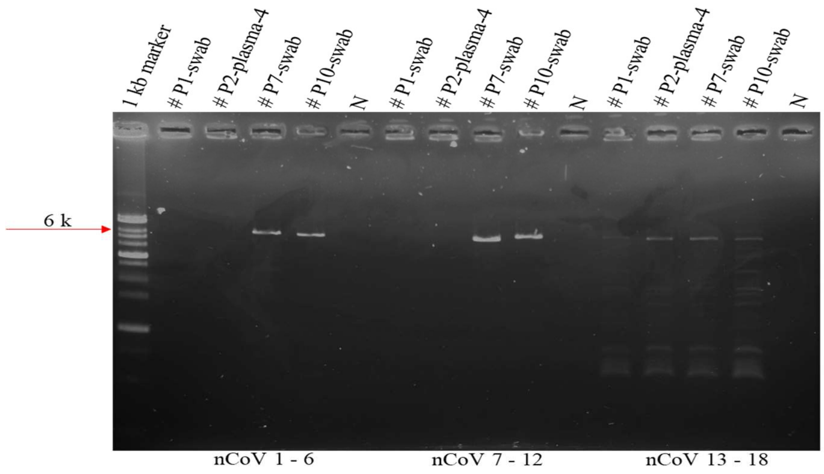

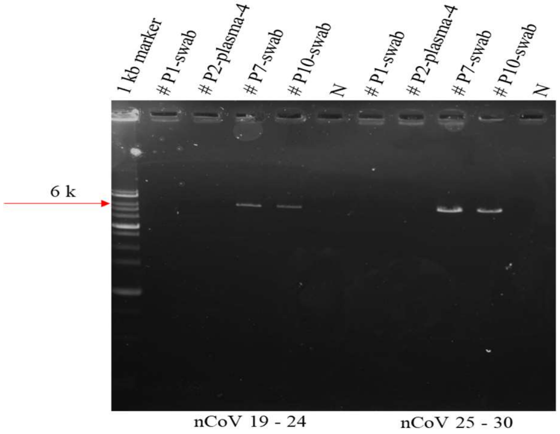

Figure S1. The results of the PCR products are loaded directly onto 1% agarose TBE gels after in-house RT-PCR. The loading volume for each lane is 4.5 ul (sample: dye = 9: 1) and uses a 1 kb DNA ladder as a marker. Whole genome sequences are separated into 5 fragments (≈ 6 kb each). The location of all fragments is as follows: Fragment 1 (nCoV 1-6: 1 - 6078), Fragment 2 (nCoV 7-12: 6107 - 12089), Fragment 3 (nCoV 13-18: 11998 - 18101), Fragment 4 (nCoV 19-24: 18019 - 24096), and Fragment 5 (nCoV 25-30: 24009 - 29837).

	18	20	26	49	67	68	69	70	74	80	94	138	144	145	188	189	190	215
**NC045512 CHN/Wuhan-Hu-1 B**	**L**	**T**	**P**	**H**	**A**	**I**	**H**	**V**	**N**	**D**	**S**	**D**	**Y**	**Y**	**N**	**L**	**R**	**D**
Consensus B	.	.	.	—	.	.	.	.	—	.	.	.	.	.	.	.	.	.
Consensus B_TWN only	.	.	.	—	.	.	.	—	.	.	.	.	.	.	.	.	.	.
TWN/NYCU-P7	.	.	.	.	.	.	.	.	.	.	.	.	.	.	.	.	.	.
TWN/NYCU-P10	.	.	.	.	.	.	.	.	.	.	.	.	.	.	.	.	.	.
Consensus Alpha	.	.	.	.	.	—	—	—	.	.	—	.	—	—	.	.	.	.
Consensus Alpha_TWN only	.	.	.	.	.	—	—	—	.	.	.	.	—	—	.	.	.	.
Consensus Beta	F	.	.	.	.	.	.	.	.	A	.	.	.	.	.	.	.	G
Consensus Beta_TWN only	F	.	.	.	V	.	.	.	.	A	.	.	.	.	.	.	.	G
Consensus Gamma	F	N	S	.	.	.	.	.	.	.	.	Y	.	.	.	.	S	.
Consensus Gamma_TWN only	F	N	S	.	.	.	.	.	.	.	.	Y	.	.	—	—	—	.

	221	242	243	244	245	416	417	484	501	570	614	655	677	681	682	701	716	797
**NC045512 CHN/Wuhan-Hu-1 B**	**S**	**L**	**A**	**L**	**H**	**G**	**K**	**E**	**N**	**A**	**D**	**H**	**Q**	**P**	**R**	**A**	**T**	**F**
Consensus B	.	.	.	.	.	.	.	.	.	.	.	.	.	.	.	.	.	—
Consensus B_TWN only	.	.	.	.	.	.	.	.	.	.	.	.	.	.	.	.	.	.
TWN/NYCU-P7	.	.	.	.	.	.	.	.	.	.	.	.	.	.	.	.	.	.
TWN/NYCU-P10	.	.	.	.	.	.	.	.	.	.	.	.	.	.	.	.	.	.
Consensus Alpha	.	.	.	.	.	.	.	.	Y	—	G	.	.	H	.	.	I	.
Consensus Alpha_TWN only	—	.	.	.	.	—	.	—	Y	D	G	.	.	H	.	.	I	.
Consensus Beta	.	—	—	—	—	.	—	K	Y	.	G	.	—	.	—	V	.	.
Consensus Beta_TWN only	.	—	—	—	—	.	N	K	Y	.	G	.	.	.	.	V	.	.
Consensus Gamma	.	.	.	.	.	.	T	K	Y	.	G	Y	.	.	.	.	.	.
Consensus Gamma_TWN only	.	.	.	.	.	.	T	K	Y	.	G	Y	.	.	.	.	.	.

	884	982	1027	1118	1176	1177
**NC045512 CHN/Wuhan-Hu-1 B**	**S**	**S**	**T**	**D**	**V**	**N**
Consensus B	.	.	.	.	.	.
Consensus B_TWN only	—	.	.	.	.	.
TWN/NYCU-P7	.	.	.	.	.	.
TWN/NYCU-P10	.	.	.	.	.	.
Consensus Alpha	.	A	.	H	.	—
Consensus Alpha_TWN only	.	A	.	H	.	.
Consensus Beta	.	.	.	.	.	.
Consensus Beta_TWN only	.	.	.	.	.	.
Consensus Gamma	.	.	I	.	F	.
Consensus Gamma_TWN only	.	.	I	.	F	.

[a] Location from the start of the NC045512.2 nucleotide sequence 21563-25384. The total length of the Spike protein is 1273 amino acids.

b The amino acids that differ from NC045512.2 are highlighted on their own. Deletion (-) and identical (.) amino acids are indicated; discord motifs within the same group are also shaded.

Figure S2. Comparison of amino acid usage patterns in spike protein between globally recognized variant strains and early cases of SARS-CoV-2 infection in Taiwan.

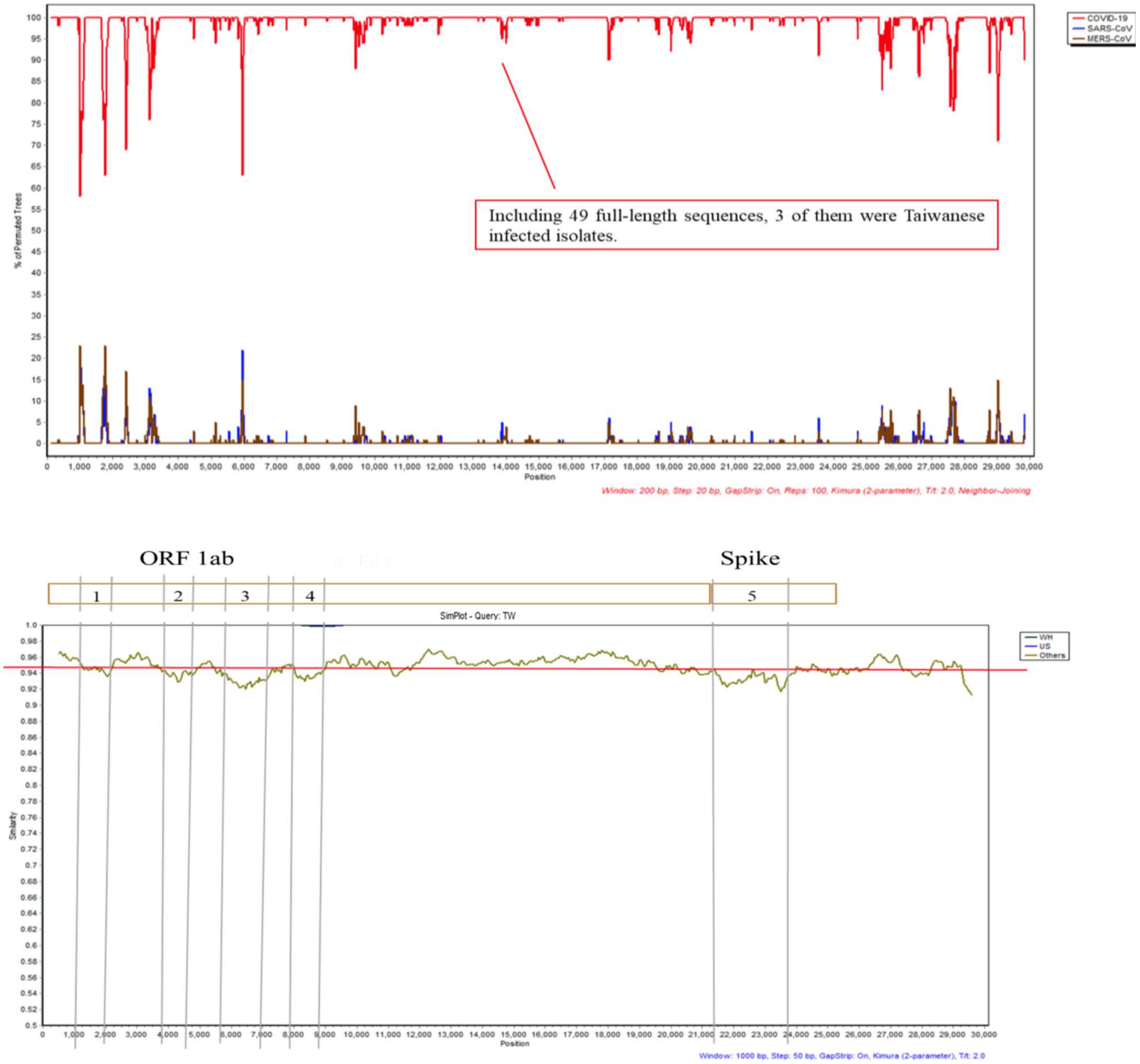

Figure S3. Using SimPlot (version 3.5.1) to determine the characteristics of the virus strain, putative recombinants, and their similarity. The upper part shows that 49 SARS-CoV-2 isolates belong to the same cluster, neither SARS-CoV nor MERS-CoV. The lower part presents 5 segments with worse similarity (< 95%, v1: 1000 - 2000, v2: 3750 - 4500, v3: 5600 - 7000, v4: 7800 - 8750, & v5: 21300 - 23750) than in other positions.

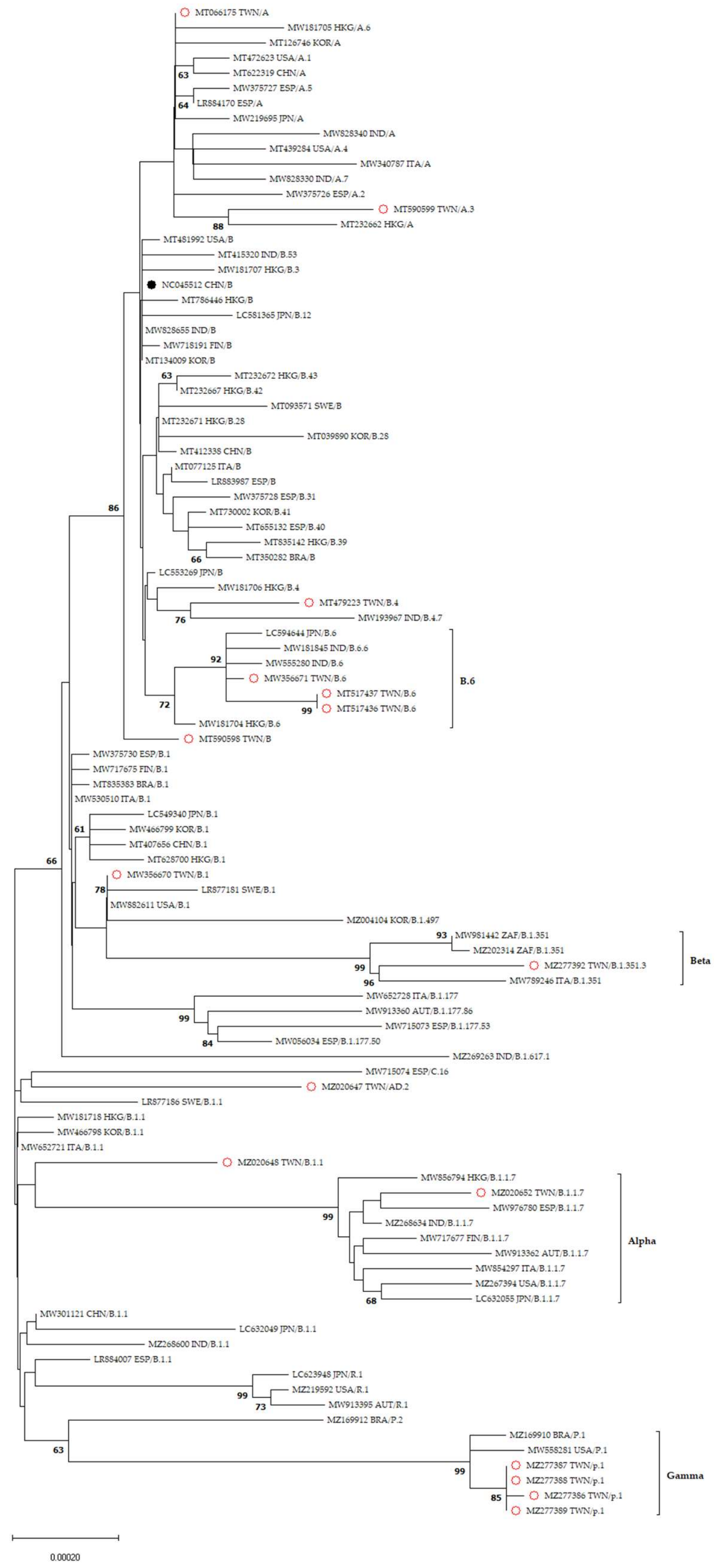

Figure S4. Phylogenetic analysis of SARS-CoV-2 strains that circulated throughout the world. There were 100 nucleotide sequences in the final dataset. Using neighbor-joining (NJ) and near full-length nucleotide sequences (29116 bp) to build an unrooted phylogenetic tree.

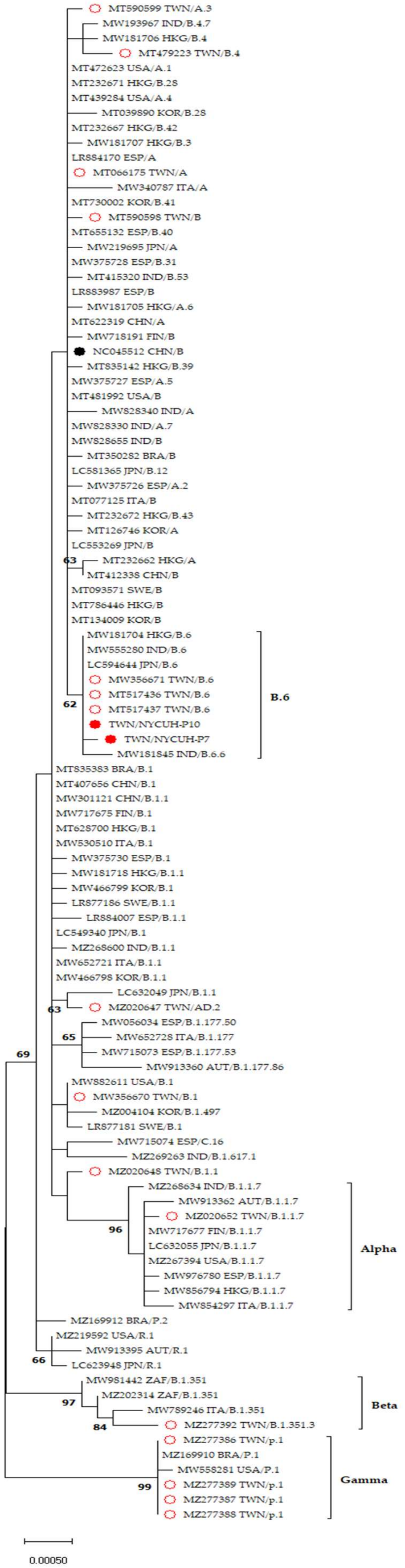
MT590599 TWN/A.3
MW193967 IND/B.4.7
MW181706 HKG/B.4
MT479223 TWN/B.4
MT472623 USA/A.1
MT232671 HKG/B.28
MT439284 USA/A.4
MT039890 KOR/B.28
MT232667 HKG/B.42
MW181707 HKG/B.3
LR884170 ESP/A
MT066175 TWN/A
MW340787 ITA/A
MT730002 KOR/B.41
MT590598 TWN/B
MT655132 ESP/B.40
MW219695 JPN/A
MW375728 ESP/B.31
MT415320 IND/B.53
LR883987 ESP/B
MW181705 HKG/A.6
MT622319 CHN/A
MW718191 FIN/B
NC045512 CHN/B
MT835142 HKG/B.39
MW375727 ESP/A.5
MT481992 USA/B
MW828340 IND/A
MW828330 IND/A.7
MW828655 IND/B
MT350282 BRA/B
LC581365 JPN/B.12
MW375726 ESP/A.2
MT077125 ITA/B
MT232672 HKG/B.43
MT126746 KOR/A
LC553269 JPN/B
63
MT232662 HKG/A
MT412338 CHN/B
MT093571 SWE/B
MT786446 HKG/B
MT134009 KOR/B
MW181704 HKG/B.6
MW555280 IND/B.6
LC594644 JPN/B.6
MW356671 TWN/B.6
62
MT517436 TWN/B.6
MT517437 TWN/B.6
TWN/NYCUH-P10
TWN/NYCUH-P7
MW181845 IND/B.6.6
B.6
MT835383 BRA/B.1
MT407656 CHN/B.1
MW301121 CHN/B.1.1
MW717675 FIN/B.1
MT628700 HKG/B.1
MW530510 ITA/B.1
MW375730 ESP/B.1
MW181718 HKG/B.1.1
MW466799 KOR/B.1
LR877186 SWE/B.1.1
LR884007 ESP/B.1.1
LC549340 JPN/B.1
MZ268600 IND/B.1.1
MW652721 ITA/B.1.1
MW466798 KOR/B.1.1
LC632049 JPN/B.1.1
63
MZ020647 TWN/AD.2
MW056034 ESP/B.1.177.50
65
MW652728 ITA/B.1.177
MW715073 ESP/B.1.177.53
69
MW913360 AUT/B.1.177.86
MW882611 USA/B.1
MW356670 TWN/B.1
MZ004104 KOR/B.1.497
LR877181 SWE/B.1
MW715074 ESP/C.16
MZ269263 IND/B.1.617.1
MZ020648 TWN/B.1.1
MZ268634 IND/B.1.1.7
MW913362 AUT/B.1.1.7
MZ020652 TWN/B.1.1.7
96
MW717677 FIN/B.1.1.7
LC632055 JPN/B.1.1.7
Alpha
MZ267394 USA/B.1.1.7
MW976780 ESP/B.1.1.7
MW856794 HKG/B.1.1.7
MW854297 ITA/B.1.1.7
MZ169912 BRA/P.2
MZ219592 USA/R.1
MW913395 AUT/R.1
66
LC623948 JPN/R.1
MW981442 ZAF/B.1.351
MZ202314 ZAF/B.1.351
97
MW789246 ITA/B.1.351
Beta
84
MZ277392 TWN/B.1.351.3
MZ277386 TWN/p.1
MZ169910 BRA/P.1
MW558281 USA/P.1
Gamma
99
MZ277389 TWN/p.1
MZ277387 TWN/p.1
MZ277388 TWN/p.1
0.00050

Figure S5a

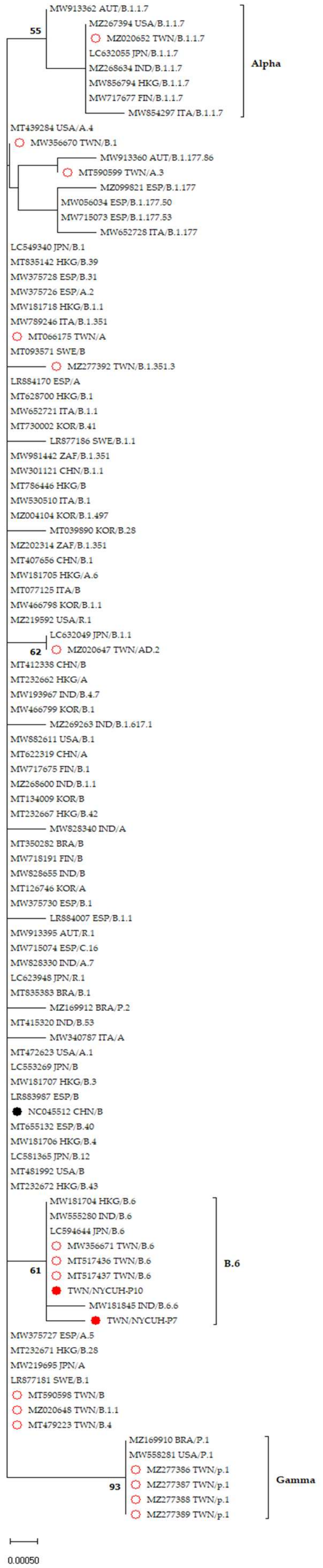
MW913362 AUT/B.1.1.7
MZ267394 USA/B.1.1.7
MZ020652 TWN/B.1.1.7
LC632055 JPN/B.1.1.7
MZ268634 IND/B.1.1.7
MW856794 HKG/B.1.1.7
MW717677 FIN/B.1.1.7
MW854297 ITA/B.1.1.7
55
Alpha
MT439284 USA/A.4
MW356670 TWN/B.1
MW913360 AUT/B.1.177.86
MT590599 TWN/A.3
MZ099821 ESP/B.1.177
MW056034 ESP/B.1.177.50
MW715073 ESP/B.1.177.53
MW652728 ITA/B.1.177
LC549340 JPN/B.1
MT835142 HKG/B.39
MW375728 ESP/B.31
MW375726 ESP/A.2
MW181718 HKG/B.1.1
MW789246 ITA/B.1.351
MT066175 TWN/A
MT093571 SWE/B
MZ277392 TWN/B.1.351.3
LR884170 ESP/A
MT628700 HKG/B.1
MW652721 ITA/B.1.1
MT730002 KOR/B.41
LR877186 SWE/B.1.1
MW981442 ZAF/B.1.351
MW301121 CHN/B.1.1
MT786446 HKG/B
MW530510 ITA/B.1
MZ004104 KOR/B.1.497
MT039890 KOR/B.28
MZ202314 ZAF/B.1.351
MT407656 CHN/B.1
MW181705 HKG/A.6
MT077125 ITA/B
MW466798 KOR/B.1.1
MZ219592 USA/R.1
LC632049 JPN/B.1.1
MZ020647 TWN/AD.2
62
MT412338 CHN/B
MT232662 HKG/A
MW193967 IND/B.4.7
MW466799 KOR/B.1
MZ269263 IND/B.1.617.1
MW882611 USA/B.1
MT622319 CHN/A
MW717675 FIN/B.1
MZ268600 IND/B.1.1
MT134009 KOR/B
MT232667 HKG/B.42
MW828340 IND/A
MT350282 BRA/B
MW718191 FIN/B
MW828655 IND/B
MT126746 KOR/A
MW375730 ESP/B.1
LR884007 ESP/B.1.1
MW913395 AUT/R.1
MW715074 ESP/C.16
MW828330 IND/A.7
LC623948 JPN/R.1
MT835383 BRA/B.1
MZ169912 BRA/P.2
MT415320 IND/B.53
MW340787 ITA/A
MT472623 USA/A.1
LC553269 JPN/B
MW181707 HKG/B.3
LR883987 ESP/B
NC045512 CHN/B
MT655132 ESP/B.40
MW181706 HKG/B.4
LC581365 JPN/B.12
MT481992 USA/B
MT232672 HKG/B.43
MW181704 HKG/B.6
MW555280 IND/B.6
LC594644 JPN/B.6
MW356671 TWN/B.6
MT517436 TWN/B.6
MT517437 TWN/B.6
TWN/NYCUH-P10
MW181845 IND/B.6.6
TWN/NYCUH-P7
61
B.6
MW375727 ESP/A.5
MT232671 HKG/B.28
MW219695 JPN/A
LR877181 SWE/B.1
MT590598 TWN/B
MZ020648 TWN/B.1.1
MT479223 TWN/B.4
MZ169910 BRA/P.1
MW558281 USA/P.1
MZ277386 TWN/p.1
MZ277387 TWN/p.1
MZ277388 TWN/p.1
MZ277389 TWN/p.1
93
Gamma
0.00050

Figure S5b

Figure S5. Phylogenetic analysis of SARS-CoV-2 strains that circulated throughout the world. There were 102 nucleotide sequences in the final dataset. Using different lengths to build unrooted phylogenetic trees. Maximum likelihood (ML) trees based on the proposed method a) assembled sequence of all variant regions (v1 - v5, 6274 bp) and b) partial sequence of Nsp3/PLpro (v3 only, 1401 bp) aligned by the current study.
